# Towards an enhanced understanding of osteoanabolic effects of PTH-induced microRNAs on osteoblasts using a bioinformatic approach

**DOI:** 10.3389/fendo.2024.1380013

**Published:** 2024-07-17

**Authors:** Lucija Ana Vrščaj, Janja Marc, Barbara Ostanek

**Affiliations:** ^1^ Department of Clinical Biochemistry, Faculty of Pharmacy, University of Ljubljana, Ljubljana, Slovenia; ^2^ Clinical Institute of Clinical Chemistry and Biochemistry, University Clinical Centre Ljubljana, Ljubljana, Slovenia

**Keywords:** osteoporosis, miRNA, signaling pathways, Hippo signaling pathway, TGF-β signaling pathway, signaling pathways regulating pluripotency of stem cells, osteoblasts

## Abstract

In this study, we used a bioinformatic approach to construct a miRNA-target gene interaction network potentially involved in the anabolic effect of parathyroid hormone analogue teriparatide [PTH (1–34)] on osteoblasts. We extracted a dataset of 26 microRNAs (miRNAs) from previously published studies and predicted miRNA target interactions (MTIs) using four software tools: DIANA, miRWalk, miRDB, and TargetScan. By constructing an interactome of PTH-regulated miRNAs and their predicted target genes, we elucidated signaling pathways regulating pluripotency of stem cells, the Hippo signaling pathway, and the TGF-beta signaling pathway as the most significant pathways in the effects of PTH on osteoblasts. Furthermore, we constructed intersection of MTI networks for these three pathways and added validated interactions. There are 8 genes present in all three selected pathways and a set of 18 miRNAs are predicted to target these genes, according to literature data. The most important genes in all three pathways were *BMPR1A*, *BMPR2* and *SMAD2* having the most interactions with miRNAs. Among these miRNAs, only miR-146a-5p and miR-346 have validated interactions in these pathways and were shown to be important regulators of these pathways. In addition, we also propose miR-551b-5p and miR-338–5p for further experimental validation, as they have been predicted to target important genes in these pathways but none of their target interactions have yet been verified. Our wet-lab experiment on miRNAs differentially expressed between PTH (1–34) treated and untreated mesenchymal stem cells supports miR-186–5p from the literature obtained data as another prominent miRNA. The meticulous selection of miRNAs outlined will significantly support and guide future research aimed at discovering and understanding the crucial pathways of osteoanabolic PTH-epigenetic effects on osteoblasts. Additionally, they hold potential for the discovery of new PTH target genes, innovative biomarkers for the effectiveness and safety of osteoporosis-affected treatment, as well as novel therapeutic targets.

## 1 Introduction

Osteoporosis is a metabolic bone disease that primarily affects the elderly population. It is characterized by a gradual decline in bone mass and density, resulting in fragile bones that are susceptible to fractures (1). This decrease in bone mass is caused by an imbalance between bone-building osteoblasts and bone-resorbing osteoclasts, derived from mesenchymal stem cells (MSCs) and hematopoietic progenitors, respectively (2, 3).

Osteoblasts are specialized cells that play a crucial role in bone metabolism and homeostasis. They are responsible for producing and depositing the organic matrix of bone, which consists mostly of type 1 collagen and other proteins, as well as minerals such as calcium and phosphate in the form of hydroxyapatite. Typically, they are found on the bone surface, where they form the osteoblast layer (2). MSCs that are present in the bone marrow can differentiate into mature osteoblasts under the influence of transcription factors (for example Runx2, Osx, and Dlx5) but the process is quite complex, and a lot of signaling pathways are involved in the proliferation and differentiation of osteoblasts (4). Previous studies have shown that the Wnt/β-catenin pathway is crucial in promoting osteoblast differentiation and activity (5, 6), the BMP signaling pathway and TGF-beta signaling pathway stimulate the differentiation of MSCs into osteoblasts and promote their activity (7, 8). On the other hand, the Notch signaling pathway promotes osteoblast proliferation but inhibits their further differentiation, thus maintaining a big enough pool of undifferentiated osteoblasts (9). It is recognized that parathyroid hormone (PTH) exerts a multifaceted influence on all phases of osteoblast differentiation, encompassing early commitment to the osteoblast lineage (10), the proliferation and expansion of committed osteoprogenitor cells (11), the maturation and differentiation of osteoprogenitor cells (12), and finally, the remodeling and maintenance of mature osteoblasts (13). PTH also facilitates differentiation through miR-451a and miR-6797 (14, 15), but the direct effects of PTH on miRNAs during these stages remain largely unexplored. Moreover, there is mounting evidence suggesting that PTH affects metabolic pathways, thereby impacting bone health. Bone formation, which requires substantial energy, relies on processes such as ATP production and the breakdown of fatty acids (16). Research conducted by Esen et al. demonstrated that PTH alters intracellular metabolism by promoting aerobic glycolysis in osteoblastic MC3T3-E1 cells. Additionally, these researchers observed an increase in mitochondrial oxidative phosphorylation induced by PTH, although the exact non-glucose substrate source remains unidentified (17). It has been established that fatty acids serve as vital substrates for normal bone formation, especially during the activation of WNT-LRP5 signaling for bone growth (18, 19). Furthermore, PTH has been shown to enhance the uptake of amino acids (such as proline and glutamine) by osteoblasts, thereby promoting collagen synthesis (20–22). More pathways are involved and all these need to work in harmony as any changes can lead to bone loss. Additionally, age-related differentiation of MSCs into adipocytes rather than osteoblasts is also a contributing factor for osteoporosis (23). Although antiresorptive drugs such as bisphosphonates and denosumab are commonly used to treat osteoporosis and prevent fractures, they only prevent bone loss and do not stimulate bone formation (24). An alternative approach involves the use of osteoanabolic drugs such as teriparatide, an analog of human parathyroid hormone (PTH) consisting of its first 34 amino acids, which has been shown to promote bone formation through intermittent administration (25–27). On the contrary, continuous administration of PTH can lead to bone loss, due to its effect on osteoclasts (28). While the signaling pathways involved in the proliferation and differentiation of osteoblasts have been studied extensively (4), the osteoanabolic mechanism of PTH is not as well understood. PTH targets osteoblasts by binding to the parathyroid hormone receptor type 1 (PTH1R) on their surface and thus starts a cascade of effects on signaling pathways (29). Previous studies have shown a stimulating effect of PTH on the Wnt/β-catenin pathway, RANK/RANKL/OPG pathway, MAPK/ERK signaling pathway, and PI3K-Akt signaling pathway (30–33). All these pathways are important in bone metabolism and the balance between them is vital for efficient bone formation.

Over the years, new insights into signaling pathways in bone biology have emerged due to the discovery of non-coding RNAs (4). The most researched non-coding RNAs are miRNAs, small single-stranded non-coding RNAs, consisting of approximately 22 nucleotides. They function as regulators of gene expression by targeting mRNAs and degrading them or hindering their translation (34). We anticipate that miRNAs and their target mRNAs will provide further insights into the impact of PTH on bone-related signaling pathways. To uncover the epigenetic effects of PTH on osteoblasts, primary cells responsible for bone formation, we utilized a combination of RNA sequencing and a bioinformatics-based approach.

This study aimed to gain an insight into the osteoanabolic effect of PTH through its regulation of miRNAs in osteoblasts by performing an *in silico* and *in vitro* analysis of PTH-regulated miRNAs. We compiled a selection of previously reported PTH-regulated miRNAs in osteoblasts, from which we created an interactome with their validated and potential target mRNAs. A pathway enrichment analysis was performed to identify the most significant pathways in osteoblasts affected by PTH-regulated miRNAs. The presented interactome provides a foundation for a better grasp of the osteoanabolic mechanism of action of PTH. In the final step, we performed our RNA-sequencing experiment to determine the differential expression of PTH-treated versus untreated MSCs after 21 days of osteogenic differentiation. We compared the results with previously reported PTH-regulated miRNAs in osteoblasts.

## 2 Materials and methods

### 2.1 miRNA selection

The purpose of the study was to elucidate the mechanism of action of PTH on osteoblasts in the treatment of osteoporosis. We used a dataset of studies from a previously published literature review. We only included the five studies from the initial dataset of studies, that were studying the PTH-regulated expression of miRNAs in osteoblasts.

### 2.2 Target predictions and enrichment analysis

We used four bioinformatic tools for miRNA-target predictions:

- DIANA-microT web server v5.0 (http://diana.imis.athena-innovation.gr/DianaTools/index.php) (accessed on 13 October 2023), where target prediction is done by a DIANA-microT-CDS prediction algorithm, which is the only algorithm, that also searches for matches in 5’UTR (35).
- miRWalk v 2.0 (http://mirwalk.umm.uni-heidelberg.de/) (accessed on 15 October 2023), where target prediction is done with a machine learning algorithm (36).
- miRDB (http://mirdb.org.) (accessed on 16 October 2023), where target prediction is done by MirTarget, a machine learning algorithm (37).
- TargetScanHuman v 8.0 (https://www.targetscan.org/vert_80/) (accessed on 3 November 2023), where target prediction is done by a TargetScan algorithm, which matches miRNA seed regions with 8mer, 7mer and 6mer sites in 3’UTR (38).

As these different tools use different algorithms for target predictions and the interactions are then ranked in different ways, we compared the interactions between tools and extracted the interactions, that appeared in at least 3 tools.

We used miRTarBase v8.0 (https://mirtarbase.cuhk.edu.cn/~miRTarBase/miRTarBase_2022/php/index.php) (accessed on 3 December 2023) for exploring validated miRNA-target interactions (MTIs). The validation is divided into strong experimental evidence, where a reporter assay or Western blot is used, and weak experimental evidence, where microarray or pSILAC is used. We selected only MTIs, that are supported by strong experimental evidence (39).

Networks were created with the Cytoscape tool (https://cytoscape.org) (accessed on 23 November 2023), which was also used for analyzing the created networks (40). PTH-regulated miRNAs were then analyzed for enrichment in biological pathways using miRPath v.3 (http://diana.imis.athena-innovation.gr/DianaTools/index.php) (accessed on 27 December 2023), which uses the microT-CDS algorithm to predict the dataset miRNAs’ target genes and identify biological pathways in which they are enriched. This was done with the KEGG analysis tool, where a p-value threshold of 0.05 was used (41).

### 2.3 Small RNA sequencing

Human bone-marrow derived MSCs were acquired from adult human donors (female, age: 26) and supplied by Lonza (Switzerland). MSCs were maintained in Dulbecco’s Modified Eagle Medium (DMEM, Gibco, USA) with 1000 mg/L glucose supplemented with 10% fetal bovine serum (FBS), L-glutamine and antibiotics in humidified atmosphere with 5% CO2 at 37C. Osteogenic differentiation was induced by treatment of 1-day post confluent cells with 100 nM dexamethasone, 5mM beta-glycerophosphate and 50 mg/mL ascorbic acid-2-phosphate. Media was changed every 2–3 days. We treated MSCs with osteogenic medium for 21 days to reach the stage of mature osteoblasts. From the start of osteogenic differentiation, we treated cells with 10 nM PTH (1–34) every 2–3 days as well. The addition of PTH (1–34) was executed in two ways. First, we emulated intermittent treatment, so we added PTH for 6 hours and then replenished cells with fresh osteogenic medium without PTH. We repeated this every 2–3 days. Second, we emulated continuous treatment with PTH osteogenic medium with the addition of PTH and repeated every 2–3 days. Control cells received only osteogenic medium, which was replenished every 2–3 days. After 21 days of osteogenic differentiation, we harvested cells for RNA isolation using TRIzol (Invitrogen, MA, USA). We continued with the RNA isolation using a commercially available QIAGEN miRNeasy kit (Qiagen, Hilden, Germany).

Total amounts and integrity of RNA were assessed using the RNA Nano 6000 Assay Kit of the Bioanalyzer 2100 system (Agilent Technologies, CA, USA).

The library preparation and sequencing were performed at Novogene. A total of 1 μg total RNA per sample was used as input material for the small RNA library preparation. Briefly, 3’ and 5’ adaptors were ligated to 3’ and 5’ end of small RNA, respectively. Then the first strand cDNA was synthesized after hybridazition with reverse transcription primer. The double-stranded cDNA library was generated through PCR enrichment. After purification and size selection, libraries with insertions between 18~40 bp were ready for sequencing on Illumina sequencing with SE50. The library was checked with Qubit and real-time PCR for quantification and bioanalyzer for size distribution detection. Quantified libraries were pooled and sequenced on Illumina platforms, according to effective library concentration and data amount required.

Raw data (raw reads) of fastq format were firstly processed through custom perl and python scripts. In this step, clean data (clean reads) were obtained by removing reads containing ploy-N, with 5’ adapter contaminants, without 3’ adapter or the insert tag, containing ploy A or T or G or C and low-quality reads from raw data. At the same time, Q20, Q30, and GC-content of the raw data were calculated. Then, we chose a certain range of length from clean reads to do all the downstream analyses.

The small RNA tags were mapped to reference sequence by Bowtie (42) without mismatch to analyze their expression and distribution on the reference.

Mapped small RNA tags were used to look for known miRNA. miRBase20.0 was used as reference, modified software mirdeep2 (43) and srna-tools-cli were used to obtain the potential miRNA and draw the secondary structures. Custom scripts were used to obtain the miRNA counts as well as base bias on the first position of identified miRNA with certain length and on each position of all identified miRNA respectively.

To remove tags originating from protein-coding genes, repeat sequences, rRNA, tRNA, snRNA, and snoRNA, small RNA tags were mapped to RepeatMasker, Rfam database or those types of datas from the specified species itself.

The characteristics of hairpin structure of miRNA precursor can be used to predict novel miRNA. The available software miREvo (44) and mirdeep2 were integrated to predict novel miRNA through exploring the secondary structure, the Dicer cleavage site and the minimum free energy of the small RNA tags unannotated in the former steps. At the same time, custom scripts were used to obtain the identified miRNA counts as well as base bias on the first position with certain length and on each position of all identified miRNA respectively.

In the alignment and annotation step, some small RNA tags may be mapped to more than one category. To make every unique small RNA mapped to only one annotation, we follow the following priority rule: known miRNA > rRNA > tRNA > snRNA > snoRNA > repeat > gene > NAT-siRNA > gene > novel miRNA > ta-siRNA. The total rRNA proportion was used a marker as sample quality indicator. Usually, it should be less than 60% in plant samples and 40% in animal samples as high quality.

Position 2~8 of a mature miRNA was called seed region which were highly conserved. The target of a miRNA might be different with the changing of nucleotides in this region. In our analysis pipeline, miRNA which might have base edit could be detected by aligning all the small RNA tags to mature miRNA, allowing one mismatch.

miRNA expression levels were estimated by TPM (transcript per million) through the following criteria (45):

Normalization formula:


$$Normalized\;expression=\frac{Mapped\;readcount}{Total\;reads}\;\times 10^{6}$$

Differential expression analysis of two samples was performed using the DEGseq (2010) R package. P-value was adjusted using qvalue (46) qvalue < 0.01 and |log2(foldchange)| > 1 was set as the threshold for significantly differential expression by default.

## 3 Results

### 3.1 Selection of Human miRNAs Influenced by PTH

**Figure 1:**
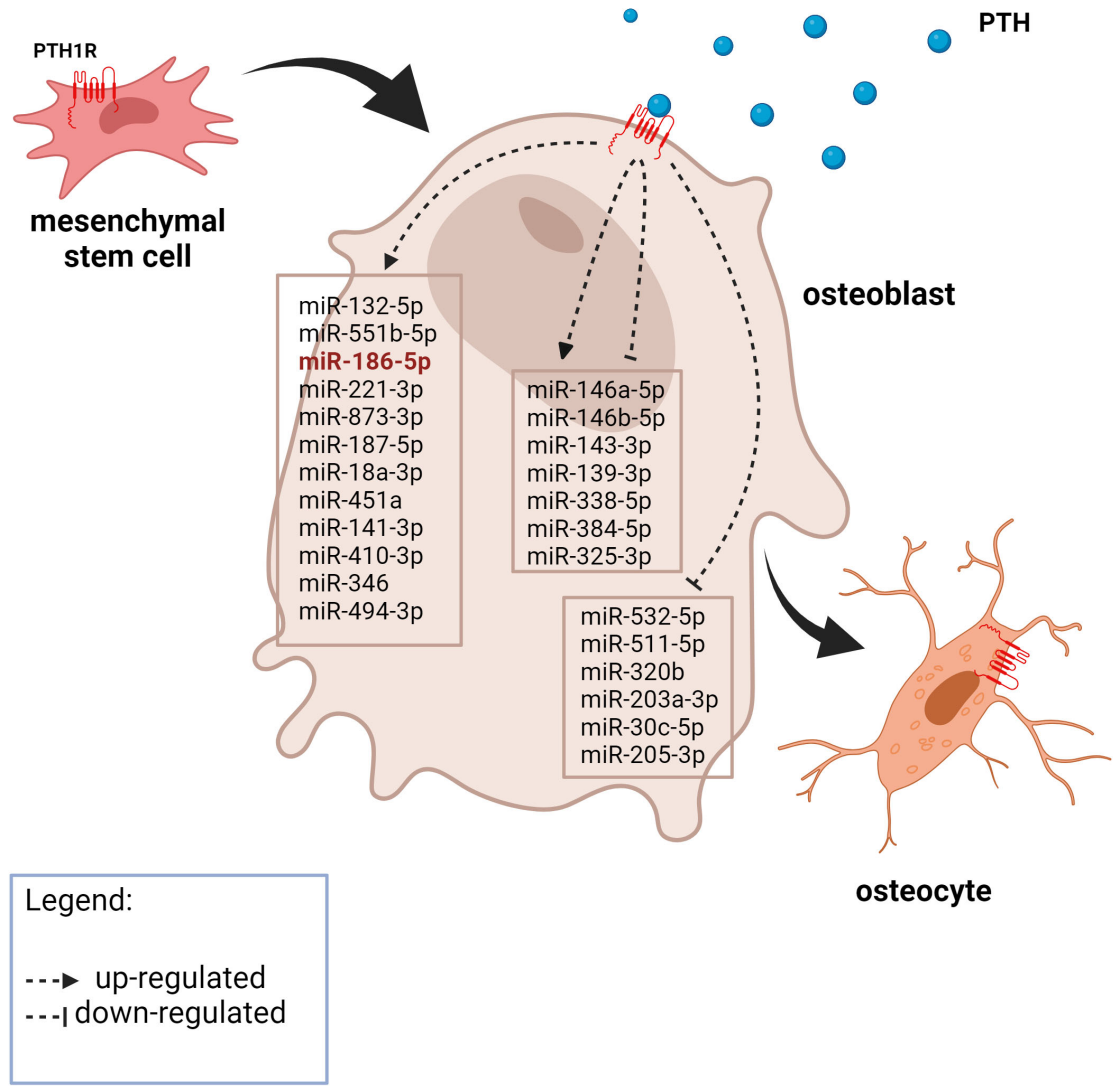
A graphic representation of the expression of PTH-related miRNAs in osteoblasts from the literature search. The miRNA in red was also significantly upregulated after 21 days of MSC osteogenic differentiation in our experiment. Created with BioRender.com.

The initial selection of miRNAs influenced by PTH was based on our previously published article (47), but for the current analysis only studies that were performed on osteoblasts were selected. These studies are compiled in **Table 1**. A summary of miRNA expression in different studies is compiled in **Table 2** and **Figure 1**.

**Table 1:** The final set and characteristics of included studies.

Author	Type of Study	miRNAs	Drug	Aim of Study
Akshaya N, et al. (48)	*in vitro* (C- rat osteoblasts)	miR-338–5p miR-384 miR-325 miR-6333 miR-290	Rat PTH (1–34)	Identify and characterize miRNAs that target Runx2 in the PTH-stimulation of MMP-13 expression in rat osteoblastic cells
Malavika D, et al. (49)	*in vitro* (C- rat osteoblasts UMR 106–01 cell line)	miR-551b-5p miR-186–5p miR-221–3p miR-873–3p miR-132–5p miR-187–5p miR-18a-3p miR-146a-5p miR-146b-5p miR-143–3p miR-139–3p	Rat PTH (1–34)	Identify and validate the functional roles of miRNAs that target HDAC4 to affect MMP-13 expression in rat osteoblasts
Karvande A, et al. (14)	*in vivo* (A-mice) + *in vitro* (C-mice osteoblasts)	miR-451a	PTH (1–34)	Evaluate PTH effects on glucose-dependent miR-451a in mice
Mohanakrishnan V, et al. (50)	*in vitro* (C- rat osteoblasts UMR 106–01 cell line)	miR-532–5p miR-511–5p miR-141–3p miR-410–3p miR-346 miR-494–3p miR-3580–5p	Rat PTH (1–34)	Evaluate PTH effects on miRNAs that target MMP-13
Laxman N, et al. (51)	*in vitro* (C-human osteoblasts)	miR-30c-5p miR-203a-3p miR-205–3p miR-320b	Teriparatide	Evaluate changes in miRNA levels in human osteoblasts after treatment with teriparatide or denosumab

C, cells; A, animals; H, humans.

**Table 2:** miRNAs and their expression in osteoblasts after PTH administration.

miRNA	Effect of PTH on miRNA expression	Comments	Reference
miR-132–5p	↑		Malavika et al. (49)
miR-551b-5p	↑	Increased expression 1h, 2h, 4h, and 8h after PTH application	Malavika et al. (49)
miR-186–5p	↑		Malavika et al. (49)
miR-221–3p	↑		Malavika et al. (49)
miR-873–3p	↑	Increased expression 2h and 8h after PTH application	Malavika et al. (49)
miR-187–5p	↑		Malavika et al. (49)
miR-18a-3p	↑		Malavika et al. (49)
miR-146a-5p	↑/↓*	Decreased expression 1h and 2h after PTH application, increased expression 4h and 8h after PTH application	Malavika et al. (49)
miR-146b-5p	↑/↓*		Malavika et al. (49)
miR-143–3p	↑/↓*	Increased expression 1h, 2h, and 8h after PTH application, decreased expression 4h after PTH application	Malavika et al. (49)
miR-139–3p	↑/↓*	Increased expression 1h, 2h, and 4h after PTH application, decreased expression 8h after PTH application	Malavika et al. (49)
miR-451a	↑		Karvande et al. (14)
miR-532–5p	↓		Mohanakrishnan et al. (50)
miR-511–5p	↓		Mohanakrishnan et al. (50)
miR-141–3p	↑		Mohanakrishnan et al. (50)
miR-410–3p	↑		Mohanakrishnan et al. (50)
miR-346	↑		Mohanakrishnan et al. (50)
miR-494–3p	↑		Mohanakrishnan et al. (50)
miR-320b	↓		Laxman et al. (51)
miR-203a-3p	↓		Laxman et al. (51)
miR-30c-5p	↓		Laxman et al. (51)
miR-205–3p	↓		Laxman et al. (51)
miR-338–5p	↑/↓*	Decreased expression 1h and 2h after PTH application; increased expression 8h after PTH application	Akshaya et al. (48)
miR-384–5p	↑/↓*		Akshaya et al. (48)
miR-325–3p	↑/↓*	Decreased expression 1h and 2h after PTH application; increased expression 4h and 12h after PTH application	Akshaya et al. (48)

↑ - PTH increases miRNA expression, ↓ - PTH decreases miRNA expression, ↑/↓ - PTH increases or decreases miRNA expression in different studies, * - the effects of PTH on miRNA expression differentiate through the study.

### 3.2 Prediction of miRNA-target Interactions

We used four different bioinformatic tools for MTI predictions. MTI predictions that appeared in at least 3 tools were used for creating a network using the Cytoscape tool.

We also used the Cytoscape tool to analyze the created network. The miRNAs had an enormous amount of interactions, e.g. miR-146a-5p had 7493 interactions and 8 miRNAs had more than a thousand interactions. The miRNAs with the most interactions were miR-146a-5p, miR-551b-5p, miR-205–3p, miR-338–5p and miR-511–5p. *DGKH* and *INO80D* were the target genes of the most miRNAs with 14 interactions and were followed by *NFIB*, *ACVR2B*, and *AGO3*, which were the target of 12, 11, and 11 miRNAs, respectively. We were not able to identify any smaller subnetworks.

### 3.3 KEGG pathway enrichment analysis

We performed a KEGG pathway enrichment analysis on all 26 miRNAs and the predicted genes with the miRPath 3.0 tool. The results are displayed in **Table 3**.

**Table 3:** Enriched pathways from the dataset. A total of 26 miRNAs were enriched in 53 pathways.

KEGG pathway	p-value	Number of genes	Number of miRNAs
Renal cell carcinoma (hsa05211)	1.25924E-09	48	24
Proteoglycans in cancer (hsa05205)	2.0867E-08	108	24
Signaling pathways regulating pluripotency of stem cells (hsa04550)	6.09134E-08	84	21
Mucin type O-Glycan biosynthesis (hsa00512)	2.05291E-07	18	13
Hippo signaling pathway (hsa04390)	4.18173E-07	81	21
Prion diseases (hsa05020)	4.64518E-07	15	14
Adherens junction (hsa04520)	1.05553E-05	47	19
Axon guidance (hsa04360)	1.27573E-05	73	22
TGF-beta signaling pathway (hsa04350)	5.91744E-05	48	19
Rap1 signaling pathway (hsa04015)	9.40696E-05	111	22
PI3K-Akt signaling pathway (hsa04151)	9.40696E-05	168	25
Circadian rhythm (hsa04710)	0.000107691	24	19
FoxO signaling pathway (hsa04068)	0.000235358	76	23
Pathways in cancer (hsa05200)	0.000314524	191	25
Ubiquitin-mediated proteolysis (hsa04120)	0.000462227	74	22
Wnt signaling pathway (hsa04310)	0.000464473	76	22
Ras signaling pathway (hsa04014)	0.000796238	109	24
Prostate cancer (hsa05215)	0.000803988	51	23
ErbB signaling pathway (hsa04012)	0.000984776	51	25
Focal adhesion (hsa04510)	0.001414196	106	24
Choline metabolism in cancer (hsa05231)	0.001632741	57	24
Estrogen signaling pathway (hsa04915)	0.002542293	46	23
Glioma (hsa05214)	0.002640411	35	21
Melanoma (hsa05218)	0.002805751	42	20
Long-term potentiation (hsa04720)	0.003144563	40	18
Prolactin signaling pathway (hsa04917)	0.003369321	38	22
Bacterial invasion of epithelial cells (hsa05100)	0.003767233	43	20
AMPK signaling pathway (hsa04152)	0.004142992	66	22
Endometrial cancer (hsa05213)	0.004910106	31	23
Regulation of actin cytoskeleton (hsa04810)	0.00563019	107	24
Long-term depression (hsa04730)	0.0057787	34	19
Protein processing in endoplasmic reticulum (hsa04141)	0.006970548	78	21
Glutamatergic synapse (hsa04724)	0.006970548	58	21
Thyroid hormone signaling pathway (hsa04919)	0.008375563	62	20
Pancreatic cancer (hsa05212)	0.008375563	36	20
ECM-receptor interaction (hsa04512)	0.00988655	38	20
Amphetamine addiction (hsa05031)	0.010669302	35	19
Adrenergic signaling in cardiomyocytes (hsa04261)	0.013140145	68	22
MAPK signaling pathway (hsa04010)	0.013371405	121	24
Non-small cell lung cancer (hsa05223)	0.014796387	29	21
Tight junction (hsa04530)	0.017538721	69	21
cGMP-PKG signaling pathway (hsa04022)	0.019348588	80	21
Oxytocin signaling pathway (hsa04921)	0.020093254	77	22
Measles (hsa05162)	0.024602787	67	19
Chronic myeloid leukemia (hsa05220)	0.027516575	38	22
HIF-1 signaling pathway (hsa04066)	0.033052144	53	20
mTOR signaling pathway (hsa04150)	0.033052144	34	20
Gap junction (hsa04540)	0.033052144	42	21
Insulin signaling pathway (hsa04910)	0.034223221	69	24
Thyroid cancer (hsa05216)	0.037130256	16	18
Oocyte meiosis (hsa04114)	0.039074828	57	20
Cholinergic synapse (hsa04725)	0.040096368	56	22
Viral carcinogenesis (hsa05203)	0.043003425	84	23

**Figure 2:**
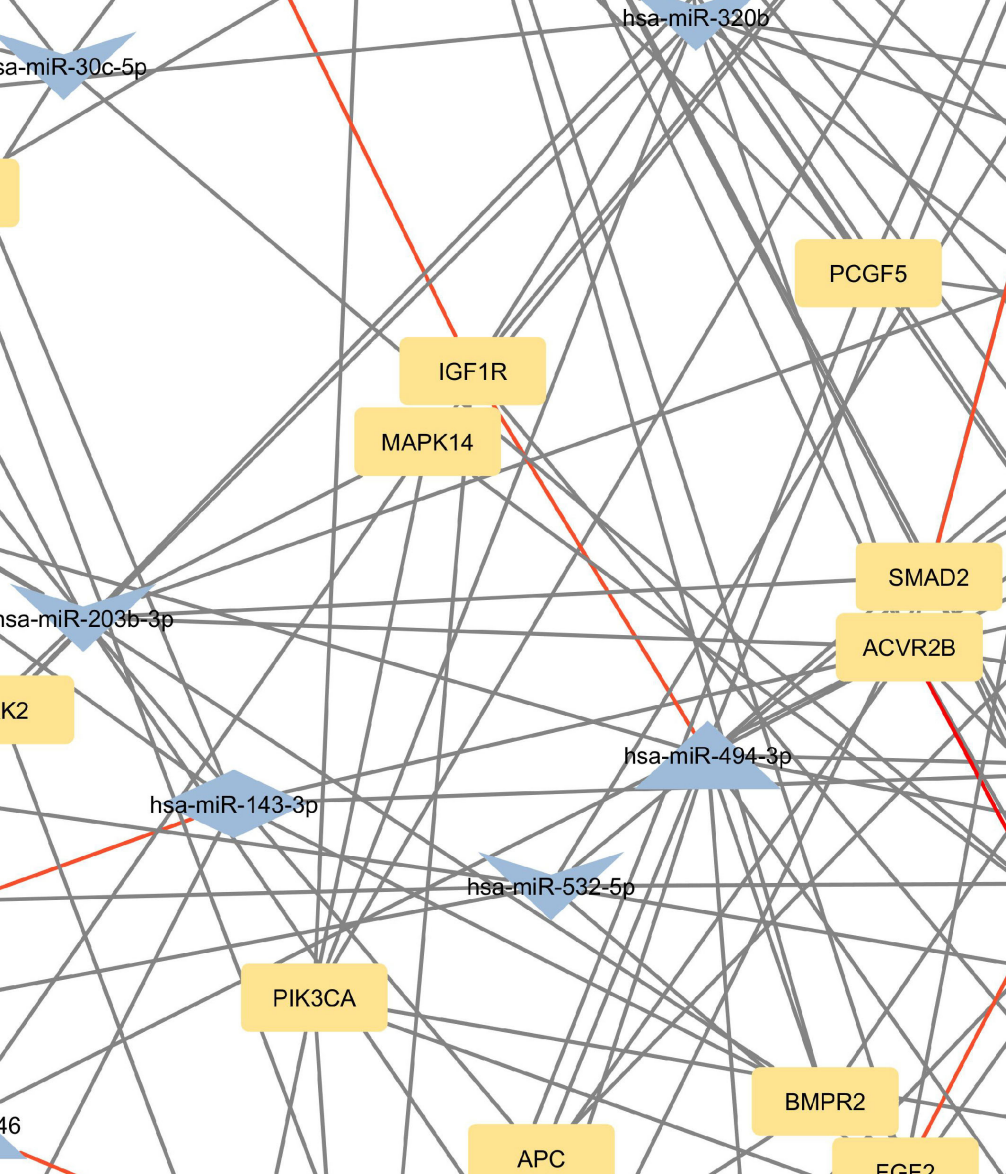
Network of predicted MTIs, enriched in signaling pathways regulating pluripotency of stem cells created with the Cytoscape software. Orange-colored nodes represent miRNAs while grey-colored nodes represent target genes. Triangle nodes represent up-regulated miRNAs after PTH administration, V-shaped nodes represent down-regulated miRNAs after PTH administration and diamond-shaped nodes represent up or down-regulated miRNAs after PTH administration. Red-colored edges represent validated interactions. The network contains 127 nodes and 295 edges. Full-size figure is available in the supplement as **Supplementary Figure S2**.

**Figure 3:**
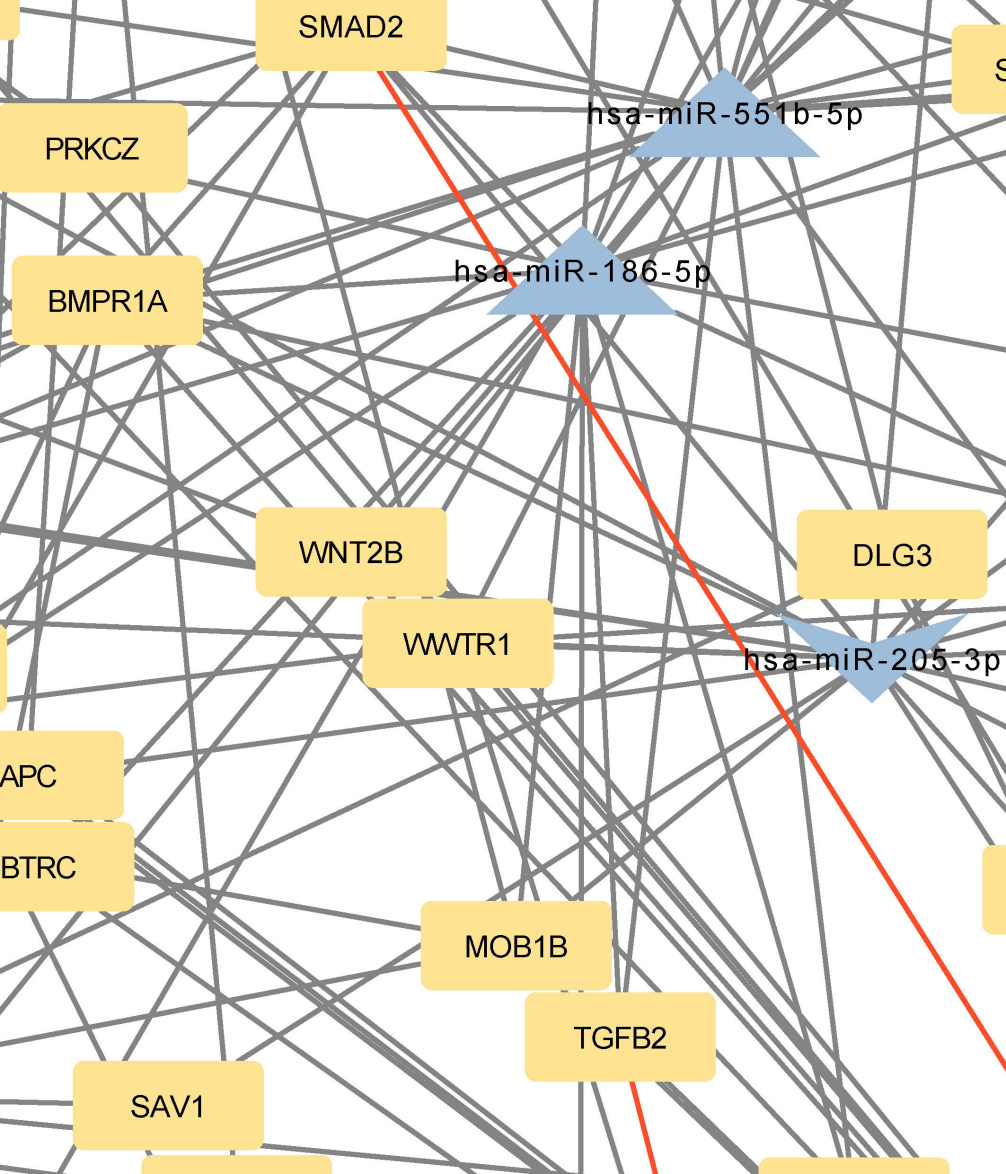
Network of predicted MTIs, enriched in Hippo signaling pathway created with the Cytoscape software. Orange-colored nodes represent miRNAs while grey-colored nodes represent target genes. Triangle nodes represent up-regulated miRNAs after PTH administration, V-shaped nodes represent down-regulated miRNAs after PTH administration and diamond-shaped nodes represent up or down-regulated miRNAs after PTH administration. Red-colored edges represent validated interactions. The network contains 131 nodes and 258 edges. Full-size figure is available in the supplement as **Supplementary Figure S3**.

**Figure 4:**
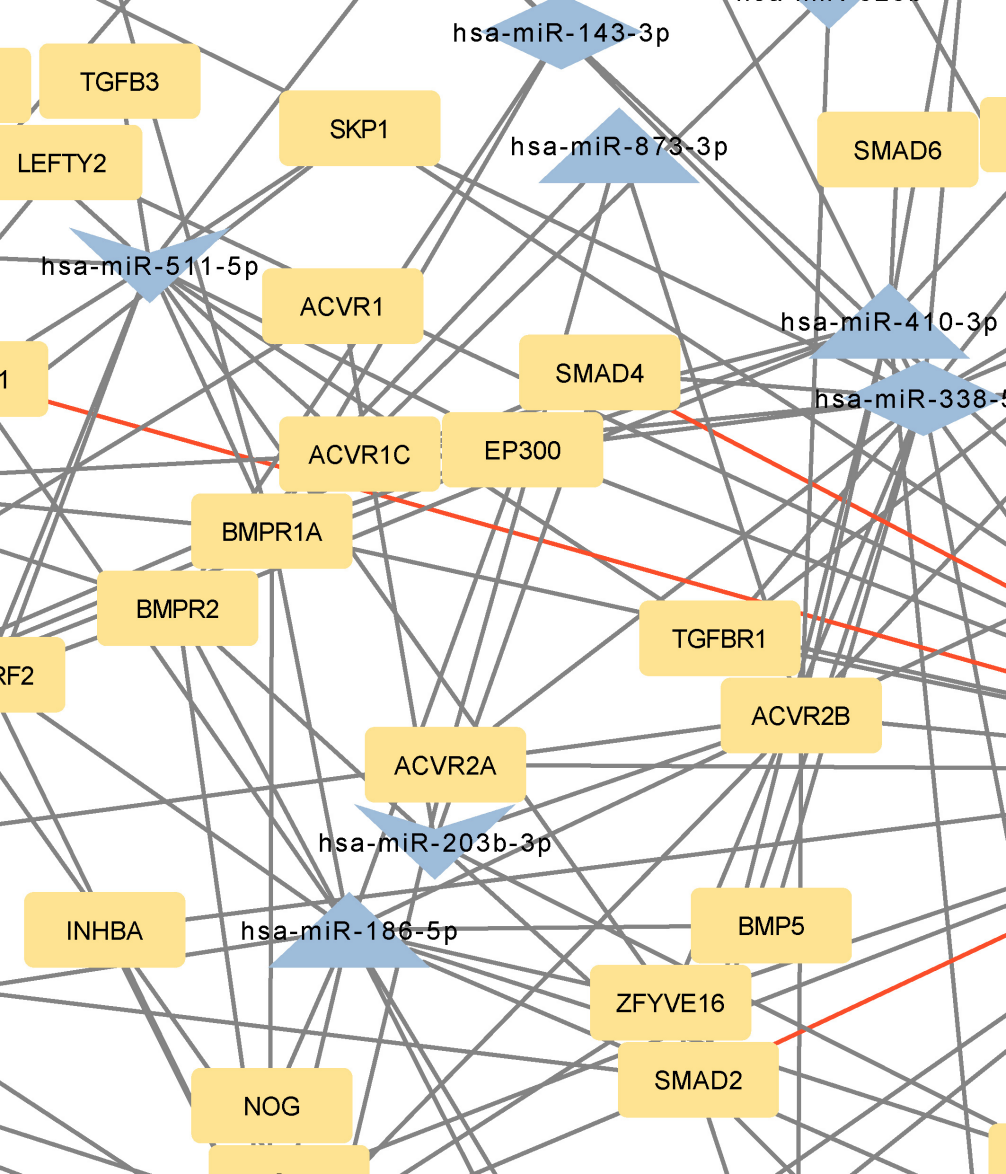
Network of predicted MTIs, enriched in TGF-beta signaling pathway created with the Cytoscape software. Orange-colored nodes represent miRNAs while grey-colored nodes represent target genes. Triangle nodes represent up-regulated miRNAs after PTH administration, V-shaped nodes represent down-regulated miRNAs after PTH administration and diamond-shaped nodes represent up or down-regulated miRNAs after PTH administration. Red-colored edges represent validated interactions. The network contains 84 nodes and 174 edges. Full-size figure is available in the supplement as **Supplementary Figure S4**.

After the analysis we manually selected three pathways, that are known to play a part in bone development and have a low p-value. These pathways were: signaling pathways regulating the pluripotency of stem cells, the Hippo signaling pathway, and the TGF-beta signaling pathway. We used Cytoscape to create visual networks of these signaling pathways (**Figures 2**–**4**).

### 3.4 Signaling pathways regulating pluripotency of stem cells, Hippo signaling pathway, and TGF-beta signaling pathway

Genes with the most interactions were *ACVR2B*, *BMPR1A*, *FZD3*, *SMAD2*, and *BMPR2*, which interacted with 11, 10, 9, 9, and 8 miRNAs, respectively. Only nine of these MTIs were validated in previous studies, and are presented in **Table 4**. Noteworthy miRNAs were miR-146a-5p, miR-551b-5p, miR-338–5p, miR-205–3p and miR-186–5p.

**Table 4:** Validated interactions of the most targeted genes in signaling pathways regulating pluripotency of stem cells.

Target gene symbol	Target gene name	miRNA
BMI1	Polycomb complex protein BMI-1	hsa-miR-338–5p
FGF2	Fibroblast growth factor 2	hsa-miR-205–3p
GSK3B	Glycogen Synthase Kinase 3 Beta	hsa-miR-346
IGF1R	Insulinlike growth factor1 receptor	hsa-miR-141–3p
		hsa-miR-494–3p
KRAS	Kirsten rat sarcoma virus	hsa-miR-143–3p
PIK3R1	Phosphoinositide-3-Kinase Regulatory Subunit 1	hsa-miR-221–3p
SMAD2	SMAD Family Member 2	hsa-miR-146a-5p
SMAD4	SMAD Family Member 4	hsa-miR-146a-5p

*BMPR1A*, *FZD3*, *SMAD2*, *BMPR2*, and *TEAD1* interacted with 10, 9, 9, 8, and 7 miRNAs, respectively. Twelve of these MTIs were validated in previous studies and are presented in **Table 5**. The most prominent miRNAs were hsa-miR-146a-5p, hsa-miR-551b-5p, hsa-miR-205–3p, miR-186–5p and hsa-miR-338–5p.

**Table 5:** Validated interactions of the most targeted genes in the Hippo pathway.

Target gene symbol	Target gene name	miRNA
BBC3	Bcl-2-binding component 3	hsa-miR-221–3p
SERPINE1	Plasminogen activator inhibitor-1	hsa-miR-143–3p
SMAD2	SMAD Family Member 2	hsa-miR-146a-5p
SMAD4	SMAD Family Member 4	hsa-miR-146a-5p
YAP1	Yes1 Associated Transcriptional Regulator	hsa-miR-141–3p
TGFB2	Transforming Growth Factor Beta 2	hsa-miR-141–3p
YWHAG	Tyrosine 3-Monooxygenase	hsa-miR-141–3p
GSK3B	Glycogen Synthase Kinase 3 Beta	hsa-miR-346
NKD1	NKD Inhibitor of WNT Signaling Pathway 1	hsa-miR-532–5p
CCND1	Cyclin D1	hsa-miR-146a-5p
CCND2	Cyclin D2	hsa-miR-146a-5p
SNAI2	Snail Family Transcriptional Repressor 2	hsa-miR-203a-3p

Genes with the most interactions were *ACVR2B*, *BMPR1A*, *SMAD2*, *BMPR2*, and *ZFYVE16* which interacted with 11, 10, 9, 8, and 6 miRNAs, respectively. Only four of these MTIs were validated in previous studies, and are presented in **Table 6**. Noteworthy miRNAs were miR-146a-5p, miR-551b-5p, miR-338–5p, miR-186–5p and miR-205–3p.

**Table 6:** Validated interactions of the most targeted genes in the TGF-beta pathway.

Target gene symbol	Target gene name	miRNA
TGFB2	Transforming Growth Factor Beta 2	hsa-miR-141–3p
SMAD2	SMAD Family Member 2	hsa-miR-146a-5p
SMAD4	SMAD Family Member 4	hsa-miR-146a-5p
ROCK1	Rho-associated coiled-coil containing protein kinase 1	hsa-miR-146a-5p

As is evident from the results, a lot of target genes are part of at least two pathways, that is why we merged the networks and the results are present in **Figure 4**.

The intersection of signaling pathways regulating pluripotency of stem cells and the Hippo signaling pathway contains 42 genes and 22 miRNAs, the intersection between Hippo and TGF-beta signaling pathway contains 19 genes and 23 miRNAs and the intersection between signaling pathways regulating pluripotency of stem cells and TGF-beta signaling pathway contains 20 genes and 22 miRNAs. The intersection of all signaling pathways contains only 8 genes and 18 miRNAs. These results aren’t surprising as we know, that a lot of genes are involved in several pathways. The number of the same genes in these pathways can be seen in **Table 7**.

**Table 7:** A number of the same genes in signaling pathways regulating pluripotency of stem cells, Hippo signaling pathway, and TGF-beta signaling pathway.

	Hippo signaling pathway	TGF-beta signaling pathway	Signaling pathways regulating pluripotency of stem cells
Hippo signaling pathway	155	**30**	**50**
TGF-beta signaling pathway	**30**	92	**29**
Signaling pathways regulating pluripotency of stem cells	**50**	**29**	143

The bolded numbers represent the number of the same genes between the pathways in the corresponding row and column. The numbers in the grey cells are the number of genes in the corresponding pathway.

### 3.5 Differentially expressed miRNAs from RNA-seq analysis

The studies included in our bioinformatic analysis obtained from the literature exhibit significant differences. They are not homogenous, as the experiments were performed either on animals or different cells (i.e., UMR 106–01 cell line, rat osteoblasts) and the results were also obtained after different treatment regimens. Therefore, we conducted our RNA-seq experiment on a single cell model and included only two types of treatment regimens: intermittent and continuous treatment.

The most prominent miRNAs in a differential analysis of PTH-treated and untreated MSCs after 21 days of differentiation are hsa-miR-31–3p, hsa-miR-451a, hsa-miR-887–3p, hsa-miR-193b-3p, hsa-miR-486–5p, hsa-miR-486–3p, hsa-miR-197–3p and hsa-miR-495–3p for intermittent and continuous treatment (**Table 8**). The most prominent miRNAs from a differential analysis between intermittent and continuous treatment with PTH are hsa-miR-1298–5p, hsa-miR-122–5p, hsa-miR-122b-3p, hsa-miR-375–3p and hsa-miR-3158–3p (**Table 8**). Of the prominent miRNAs from the bioinformatic analysis of literature data only hsa-miR-186–5p is significantly differentially expressed in continuous treatment with PTH (1–34), p< 0.015.

**Table 8:** **A** list of top 10 differentially expressed (DE) miRNAs between different treatments with PTH (1–34).

DE of continuous PTH versus untreated MSCs			
up	pval	down	pval
hsa-miR-1298–5p	4.50E-11	hsa-miR-31–3p	9.90E-16
hsa-miR-1271–5p	2.58E-07	hsa-miR-887–3p	9.63E-15
hsa-miR-122–5p	3.67E-07	hsa-miR-451a	1.44E-14
hsa-miR-122b-3p	3.67E-07	hsa-miR-193b-3p	9.42E-12
hsa-miR-543	4.07E-06	hsa-miR-491–5p	1.94E-11
hsa-let-7a-3p	1.59E-05	hsa-miR-486–5p	1.84E-10
hsa-miR-148b-3p	4.03E-05	hsa-miR-486–3p	2.12E-10
hsa-miR-25–5p	4.10E-05	hsa-miR-495–3p	2.74E-09
hsa-miR-340–3p	0.000149996	hsa-miR-324–5p	3.35E-09
hsa-miR-16–2-3p	0.00015071	hsa-miR-197–3p	3.55E-09
DE of intermittent PTH versus untreated MSCs			
up	pval	down	pval
hsa-miR-543	4.56E-09	hsa-miR-451a	4.93E-15
hsa-let-7a-3p	1.90E-07	hsa-miR-887–3p	1.25E-14
hsa-miR-148b-3p	6.64E-07	hsa-miR-31–3p	9.58E-14
hsa-miR-656–3p	1.32E-06	hsa-miR-193b-3p	2.79E-10
hsa-miR-1271–5p	1.77E-06	hsa-miR-197–3p	7.11E-10
hsa-miR-4775	2.54E-06	hsa-miR-502–5p	8.48E-10
hsa-miR-454–5p	2.84E-06	hsa-miR-486–5p	8.26E-09
hsa-miR-301a-5p	3.36E-06	hsa-miR-324–5p	9.93E-09
hsa-miR-16–2-3p	8.37E-06	hsa-miR-486–3p	1.21E-08
hsa-miR-323a-3p	1.04E-05	hsa-miR-491–5p	5.19E-08

## 4 Discussion

Our *in sillico* analysis focused on PTH effects on osteoblasts and showed that the effects are mediated through miRNAs, with the hsa-miR-146a-5p, hsa-miR-551b-5p, hsa-miR-338–5p, hsa-miR-205–3p, and hsa-miR-186–5p targeting the highest number of mRNAs.

**Figure 5:**
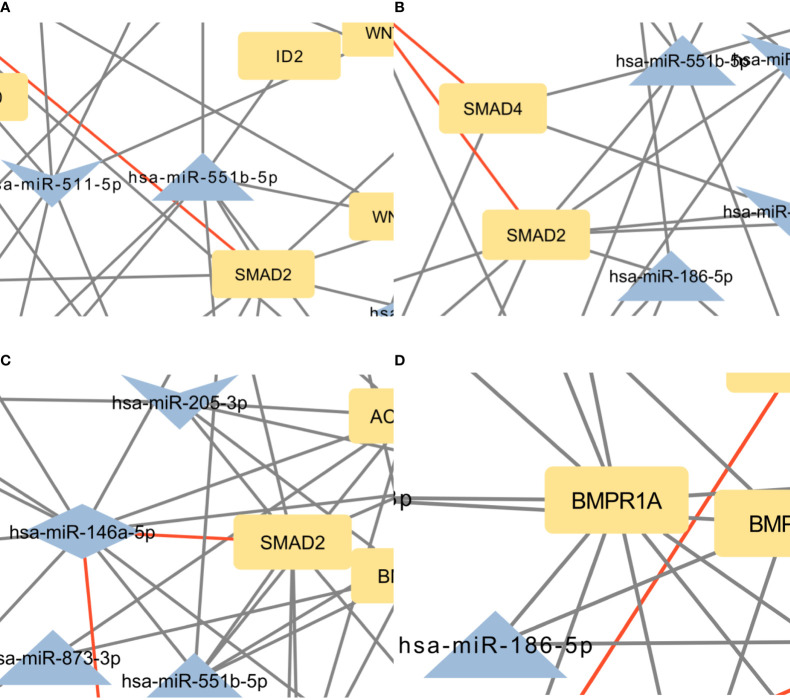
**(A)** Intersection between signaling pathways regulating pluripotency of stem cells and Hippo signaling pathway. The network contains 57 nodes and 94 edges. **(B)** Intersection between Hippo and TGF-beta signaling pathway. The network contains 42 nodes and 61 edges. **(C)** Intersection between signaling pathways regulating pluripotency of stem cells and TGF-beta signaling pathway. The network contains 44 nodes and 81 edges. **(D)** Intersection between the Hippo signaling pathway, signaling pathways regulating pluripotency of stem cells, and TGF-beta signaling pathway. The network contains 31 nodes and 39 edges. Full-size figures are available in the supplement as **Supplementary Figure S5** **(A–D)**.

The focus of the present study was on osteoblasts, which are the target cells of PTH anabolic treatments. That enabled us to identify the signaling pathways pertaining to osteoblasts. Studies, that used other samples (i.e. serum, plasma…) were excluded. The enrichment analysis for these miRNAs yielded 53 significant pathways, some of them associated with cancer and other processes, while many are important in bone development such as the signaling pathways regulating pluripotency of stem cells, the Hippo signaling pathway, the TGF-beta signaling pathway, the PI3K-Akt signaling pathway and the FoxO signaling pathway (52–57). Metabolic pathways were not significant in this dataset of miRNAs. We manually selected three signaling pathways that had the lowest p-values and are known to be involved in bone remodeling, specifically the signaling pathways regulating pluripotency of stem cells, Hippo, and TGF-beta signaling pathways. Visual networks were created, merged and the intersections analyzed. Meanwhile, we searched the miRTarBase database for all experimentally validated interactions within our dataset. miRTarBase includes the results of various validation methods, but we only selected interactions that were validated using strong experimental evidence such as Western blot and reporter assay. The red-colored edges in **Figures 2**–**5** show strong validation.

Subsequent enrichment analysis provided further insight into the dataset’s PTH-regulated miRNAs’ involvement in signaling pathways. In the Hippo signaling pathway, *SMAD2*, *FZD3*, *BMPR2*, *BMPR1A*, and *TEAD1* were the genes with the most predicted interactions. All of these genes are associated with bone formation and osteoporosis (57–60). The *SMAD2* gene encodes the SMAD family member 2 (SMAD2) protein, which is a specific mediator of the TGF-beta signaling pathway (57). Smad family members transmit signals from all receptors activated by the TGF-beta superfamily members to target genes in the nucleus (8, 61). *BMPR2* and *BMPR1A* genes encode bone morphogenic protein 2 receptors and are also part of the TGF-beta superfamily. They have potent osteogenic effects, as they are both receptors for bone morphogenic protein 2 (BMP2), an important growth factor that induces osteoblast and osteoclast activity (62). *FZD3* is a gene that encodes frizzled class receptor 3 (FZD3), which is a receptor in the WNT signaling pathway (63), an important pathway in the development of osteoporosis and part of signaling pathways regulating pluripotency of stem cells (64). FZD3 is activated in the osteogenic differentiation of bone MSCs (63). Transcription factor TEA domain family member 1 (TEAD1), encoded by the *TEAD1* gene can be linked to osteoblast and osteoclast differentiation through its coactivators YAP/TAZ, though the conclusions are controversial (65, 66). Within our network, only two miRNAs, miR-186–5p and miR-410–3p, are predicted to bind and regulate all of these genes. Of these interactions, only the interaction between hsa-miR-146a-5p and *SMAD2* is validated.

In the signaling pathways regulating pluripotency of stem cells, the most prominent genes were *ACVR2B*, *BMPR1A*, *FZD3*, *SMAD2*, and *BMPR2*. It is worth noting, that all of these genes, except *ACVR2B* were also among the most significant genes in the Hippo pathway. Interestingly, only miR-146a-5p is predicted to target all of these genes. Similar to the Hippo signaling pathway, the only validated interaction for these genes is the interaction between hsa-miR-146a-5p and *SMAD2*.

In the TGF-beta signaling pathway, the most significant predicted genes were *ACVR2B*, *BMPR2*, *BMPR1A*, *SMAD2*, and *ZFYVE16*. Of these, *SMAD2*, *BMPR2*, *and BMPR1A* were also prominent in the Hippo signaling pathway and pathways regulating the pluripotency of stem cells. *ACVR2B* encodes activin receptor type-2B, which is a part of the TGF-beta superfamily, and causes activation of SMAD2 or SMAD3 through the binding of activin A (42). *ACVR2B* is also a prominent gene in signaling pathways regulating pluripotency of stem cells. *ZFYVE16* is a protein involved in endosomal trafficking. It binds to *SMAD4*, which promotes the formation of the SMAD2/3-SMAD4 complex and controls the transcription of target genes, connected to apoptosis (43, 44). The only validated interaction for these genes, as in Hippo signaling pathway and pathways regulating pluripotency of stem cells, is the interaction between hsa-miR-146a-5p and SMAD2.

There is a significant overlap of predicted genes between pathways, indicating a high degree of interconnectedness, therefore, the networks were merged in the next step. The top five genes by the number of interactions in each pathway, were mostly prominent in the merged pathways as well. The most significant differences were *APC* and *WNT3* between Hippo and signaling pathways regulating pluripotency of stem cells, then *TGFBR1* and *TGFB2* between Hippo and TGF-beta pathways and *SMAD5* between signaling pathways regulating pluripotency of stem cells and TGF-beta signaling pathway. Previously published data has already shown the importance of the *APC* gene in osteoblast differentiation as it affects WNT and BMP signaling pathways (45). *WNT3* is known to prevent apoptosis of osteoblasts (46). *SMAD5*, a transcription factor activated by BMP2 receptors, forms a complex with SMAD4 and translocates into the nucleus to activate RUNX2 (67). RUNX2 is associated with PTH and osteoporosis and plays a crucial role in osteoblast differentiation (68, 69). There are 8 genes present in all the selected pathways and a set of 18 miRNAs are predicted to target these genes. miR-551b-5p targets 5 genes in this network, which means it could be an important miRNA in the mechanism of action of PTH on osteoblasts. Another important miRNA is miR-146a-5p as it is the single one miRNA with validated interactions with important genes in this network, namely *SMAD2* and *SMAD4*. Previously published data partly confirms this, as it was shown in a mice knockout model *in vivo* that miR-146a-5p regulates bone mass via *SIRT1* (70).

An important point in our interactome analysis is the focus on validated interactions in our networks as they are more reliable than predictions. The most relevant validated interactions are hsa-miR-146a-5p-*SMAD2* and hsa-miR-146a-5p-*SMAD4*, which are a part of all of the three most important pathways in our study. The upregulation of hsa-miR-146a-5p should downregulate *SMAD2* and *SMAD4*, which should have a negative effect on osteoblast differentiation (57). The effect of PTH on hsa-miR-146a-5p is time-dependent, so the miRNA can be either upregulated or downregulated depending on the exposure time to PTH. Short-term exposure to PTH seems to be more beneficial for osteogenic differentiation (49). Since most of the miRNAs in validated interactions are upregulated by PTH, an interesting gene is *GSK3B* because it inhibits osteoblast differentiation (71). PTH upregulates hsa-miR-346, which downregulates *GSK3B*, which should have a positive effect on osteoblast differentiation. Out of the predicted interactions, the interactions between hsa-miR-338–5p and *ID1* and hsa-miR-551b-5p and *ID2* are also interesting, because these two genes are inhibitors of differentiation (72). Both of these miRNAs are upregulated after PTH administration, so the genes could be downregulated, which promotes osteoblast differentiation. However, these interactions need to be validated.

It should be noted that the main miRNAs identified in our differential analysis differ from the main miRNAs identified in bioinformatics analysis from literature data. Only hsa-miR-186–5p was found to be significant in both analyses and was expressed differently. After PTH administration by Malavika et al. (49), hsa-miR-186–5p was up-regulated at all time points. In the RNA-seq experiment hsa-miR-186–5p was down-regulated in continuous treatment compared to untreated cells. This result shows potential, since previous studies have shown that exosomal miR-186 derived from MSCs promotes osteogenesis through the Hippo signaling pathway (73). This discrepancy between our experiment and previous studies is not surprising as the experiments differ from each other in a few aspects. As already stated, previous studies used a variety of cells with only one study using human cells. Additionally, the treatment regimens differed, with previous studies using short-term treatment, while we implemented a prolonged treatment regimen throughout the process of osteoblast differentiation. As hsa-miR186–5p is the only miRNA that is present in both the *in silico* and *in vitro* approach and thus a very promising miRNA in PTH regulation of osteoblast-related pathways.

While our focus was on signaling pathways, we explored the confirmed interactions between our miRNA dataset and critical genes involved in different stages of osteogenic differentiation (such as RUNX2, ALP, OSX, OPN, RANKL, and OPG). According to the miRTarBase database, among the top-reported miRNAs from our differential expression analysis, hsa-miR-193b-3p and hsa-miR-340–3p displayed validated interactions with RUNX2. Furthermore, hsa-miR-1271–5p and hsa-miR-324–5p demonstrated validated interactions with OSX, while hsa-miR-122–5p exhibited a validated interaction with RANKL. From the existing literature, hsa-miR-203a-3p, hsa-miR-30c-5p, and hsa-miR-320 have documented interactions with RUNX2. The limitation of our study is that our miRNA measurements were confined to the final stage of osteoblast differentiation. In future studies, measurements of miRNA expression at different stages of osteoblastogenesis would provide an even deeper insight into the mechanism of action of PTH.

It should be pointed out that the validated interactions related to osteoporosis in miRTarBase had to be manually searched for since this particular disease is not indexed in the database. This scarcity of validated interactions related to osteoporosis could be a contributing factor. In addition, miRTarBase is manually updated, which means that some validated interactions may exist in the literature but have not yet been added to the database. This may be due to the rapid pace of advancements and studies in this area, making it challenging to effectively curate such a large database.

The observed large overlap of genes through the different pathways suggests, that the dysregulation of one pathway can cause the dysregulation of a second pathway. With such a variety of pathways in which the miRNAs in the dataset are enriched, they appear to play a complex role in the mechanism of action of PTH on osteoblasts. Our analysis has shown that the most significant pathways are pathways regulating pluripotency of stem cells, Hippo signaling pathway and TGF-beta signaling pathway are influenced by PTH.

In conclusion, after *in silico* and *in vitro* analyses, we propose hsa-miR-146–5p, hsa-miR-346, hsa-miR-551b-5p, hsa-miR-338–5p and hsa-miR-186–5p as the most prominent miRNAs that could help further understand the mechanism of action of PTH on bone formation and osteoporosis. Our results show that PTH treatment effects might be mediated by these miRNAs through three main pathways, namely pathways regulating pluripotency of stem cells, Hippo signaling pathway and TGF-beta signaling pathway. Upon experimental validation, these miRNAs have the potential to serve as novel biomarkers for assessing the effectiveness of teriparatide treatment or as novel therapeutic targets in the context of osteoporosis, but further in-depth research needs to be conducted to generate more accurate conclusions.

## Data availability statement

For the preparation of the manuscript, the publicly available DIANA-microT web server v5.0 was used (http://diana.imis.athena-innovation.gr/DianaTools/index.php), accessed on 13 October 2023. miRWalk v 2.0 was used (http://mirwalk.umm.uni-heidelberg.de/), accessed on 15 October 2023. miRDB (http://mirdb.org.), accessed on 16 October 2023. TargetScanHuman v 8.0 was used (https://www.targetscan.org/vert_80/), accessed on 3 November 2023. miRTarBase v8.0 was used (https://mirtarbase.cuhk.edu.cn/~miRTarBase/miRTarBase_2022/php/index.php), accessed on 3 December 2023. Networks were created with the Cytoscape version 3.10.1. (https://cytoscape.org), accessed on 23 November 2023. miRPath v.3 was used for miRNA enrichment analysis (http://diana.imis.athena-innovation.gr/DianaTools/index.php), accessed on 27 December 2023. The sequencing data is available in the Gene Expression Omnibus (GSE259338). The original contributions presented in the study are included in the article/**Supplementary Material**. Further inquiries can be directed to the corresponding author.

## Ethics statement

Ethical approval was not required for the studies on humans in accordance with the local legislation and institutional requirements because only commercially available established cell lines were used.

## Author contributions

LV: Conceptualization, Data curation, Formal analysis, Investigation, Methodology, Validation, Visualization, Writing – original draft. JM: Conceptualization, Funding acquisition, Project administration, Resources, Supervision, Writing – review & editing. BO: Conceptualization, Funding acquisition, Project administration, Resources, Supervision, Writing – review & editing.
