# Supplementary figures and images for: Towards an enhanced understanding of osteoanabolic effects of PTH-induced microRNAs on osteoblasts using a bioinformatic approach
Source: Front Endocrinol (Lausanne). 2024 Jul 17;15:1380013. doi: 10.3389/fendo.2024.1380013 (PMC11289717; doi:10.3389/fendo.2024.1380013)

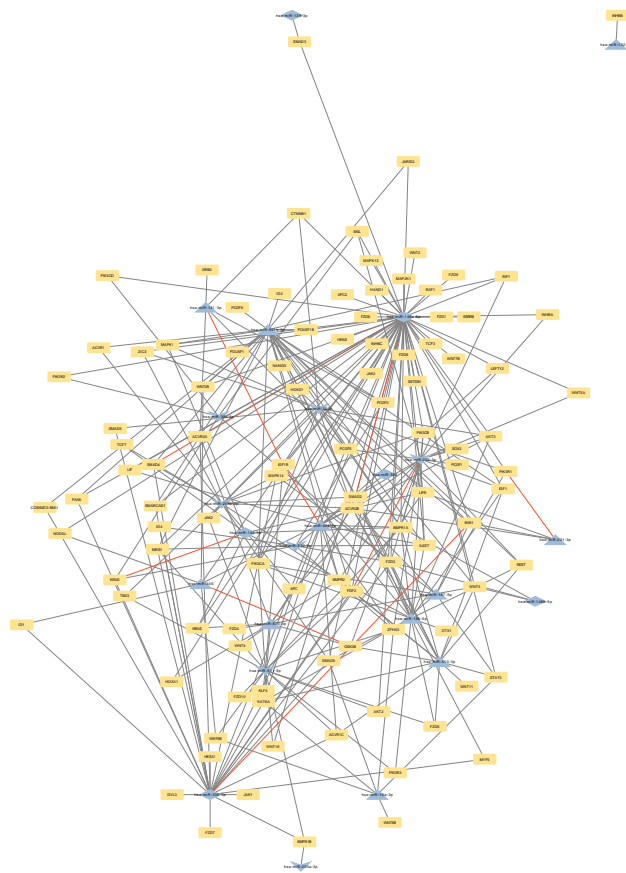

Supplement: Supplementary file 1 [file DataSheet_1.pdf]

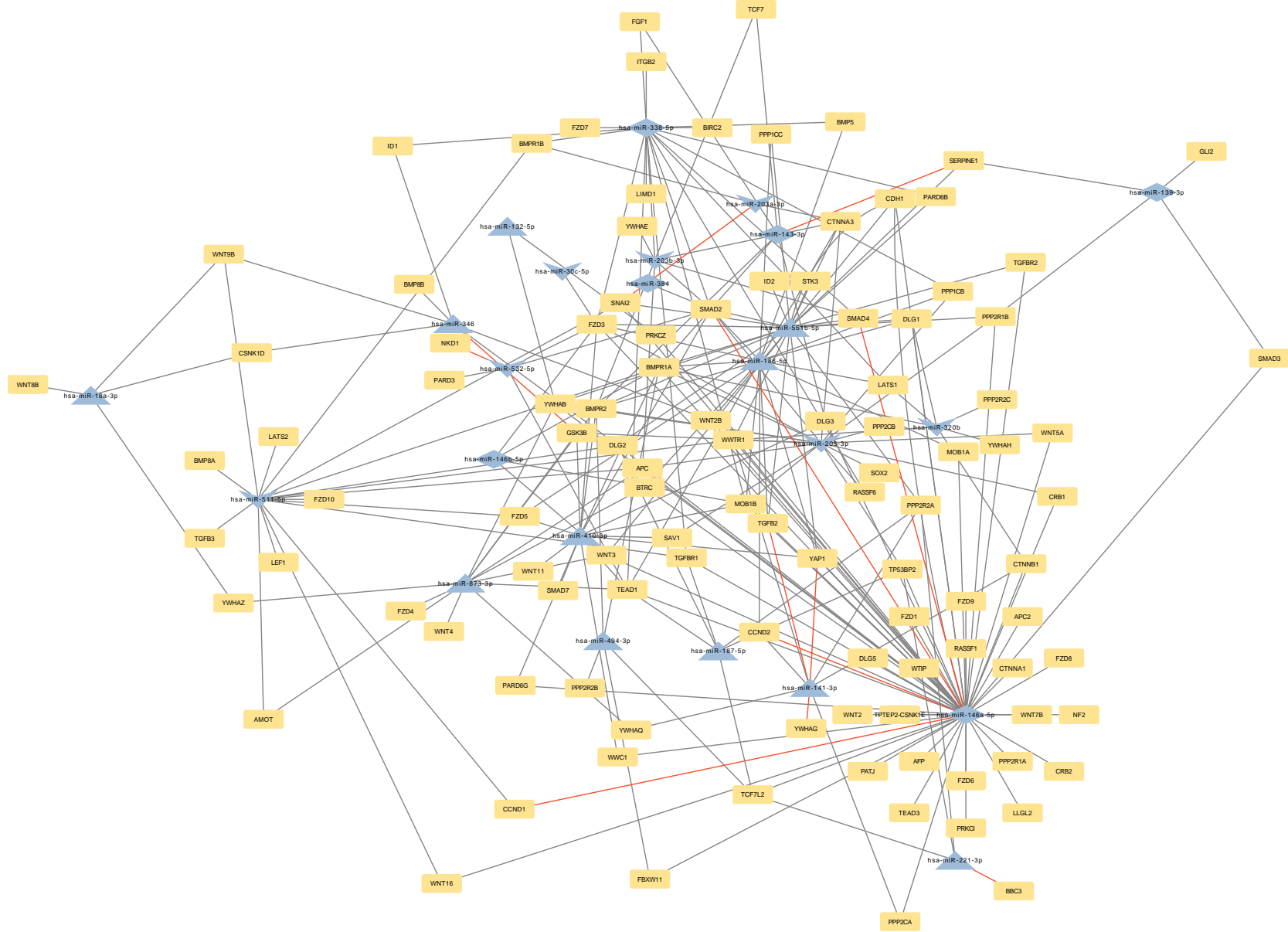

Supplement: Supplementary file 2 [file DataSheet_2.pdf]

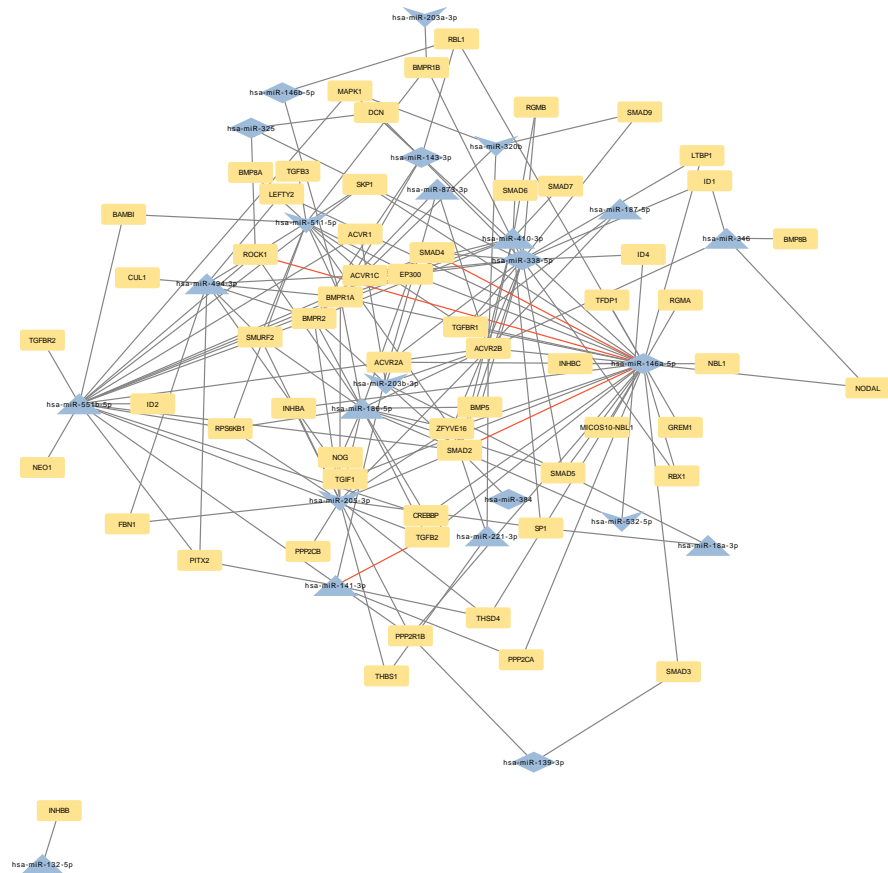

Supplement: Supplementary file 3 [file DataSheet_3.pdf]

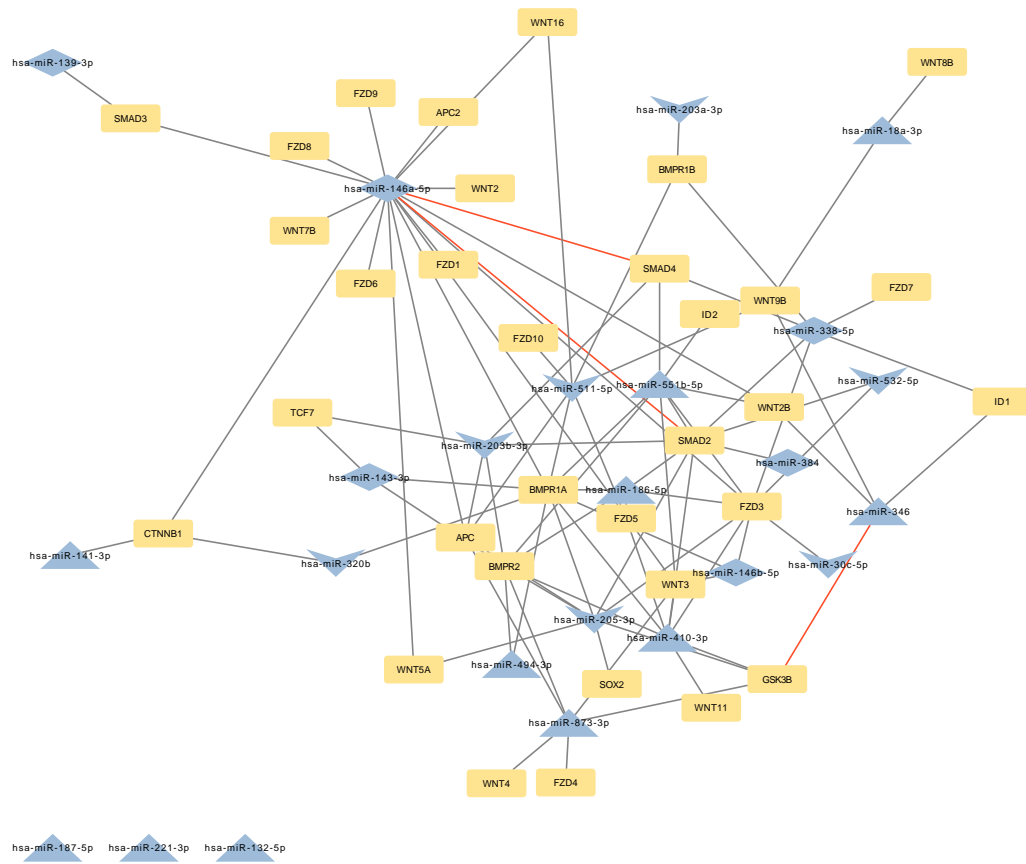

Supplement: Supplementary file 4 [file DataSheet_4.pdf]

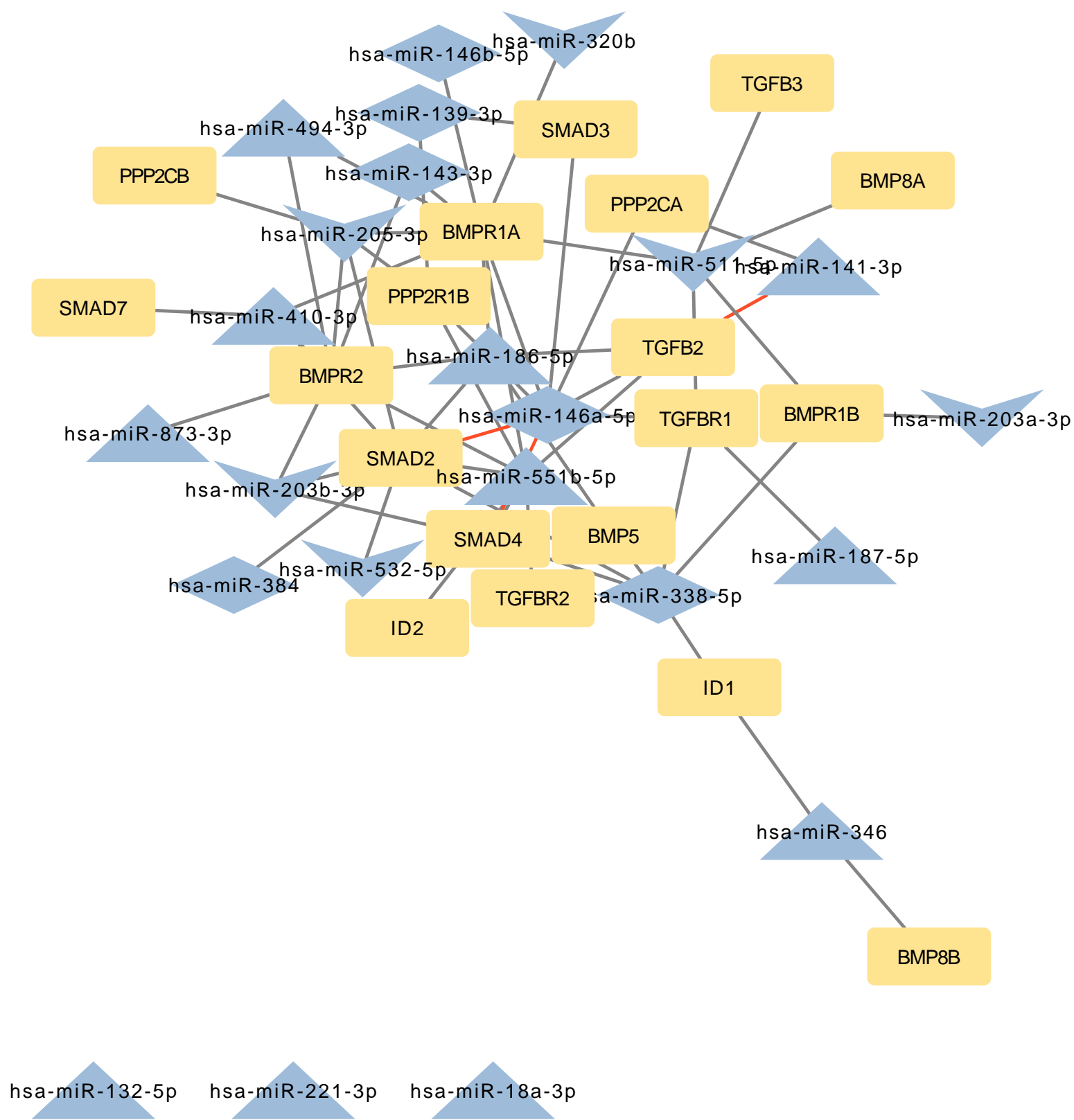

Supplement: Supplementary file 5 [file DataSheet_5.pdf]

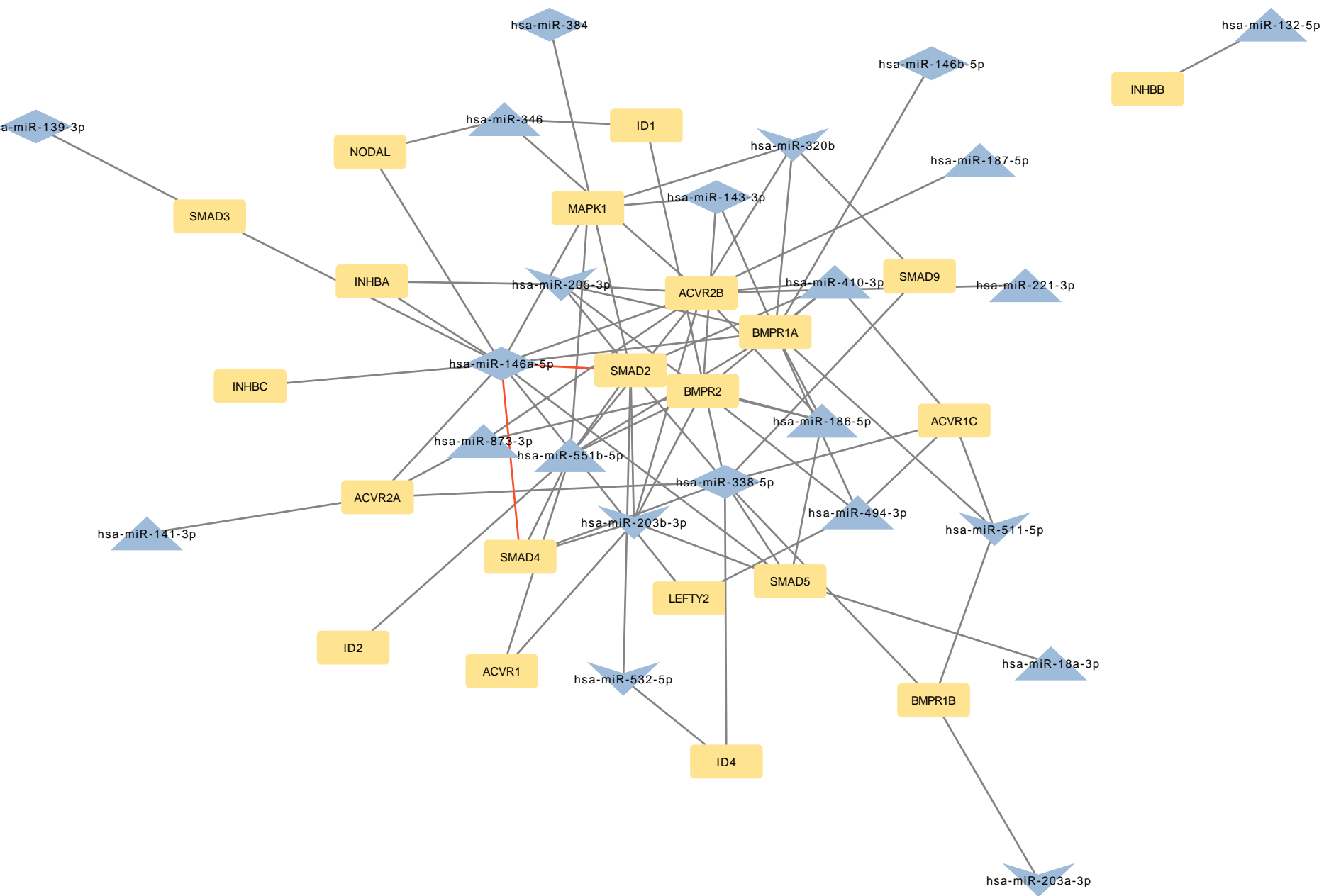

Supplement: Supplementary file 6 [file DataSheet_6.pdf]

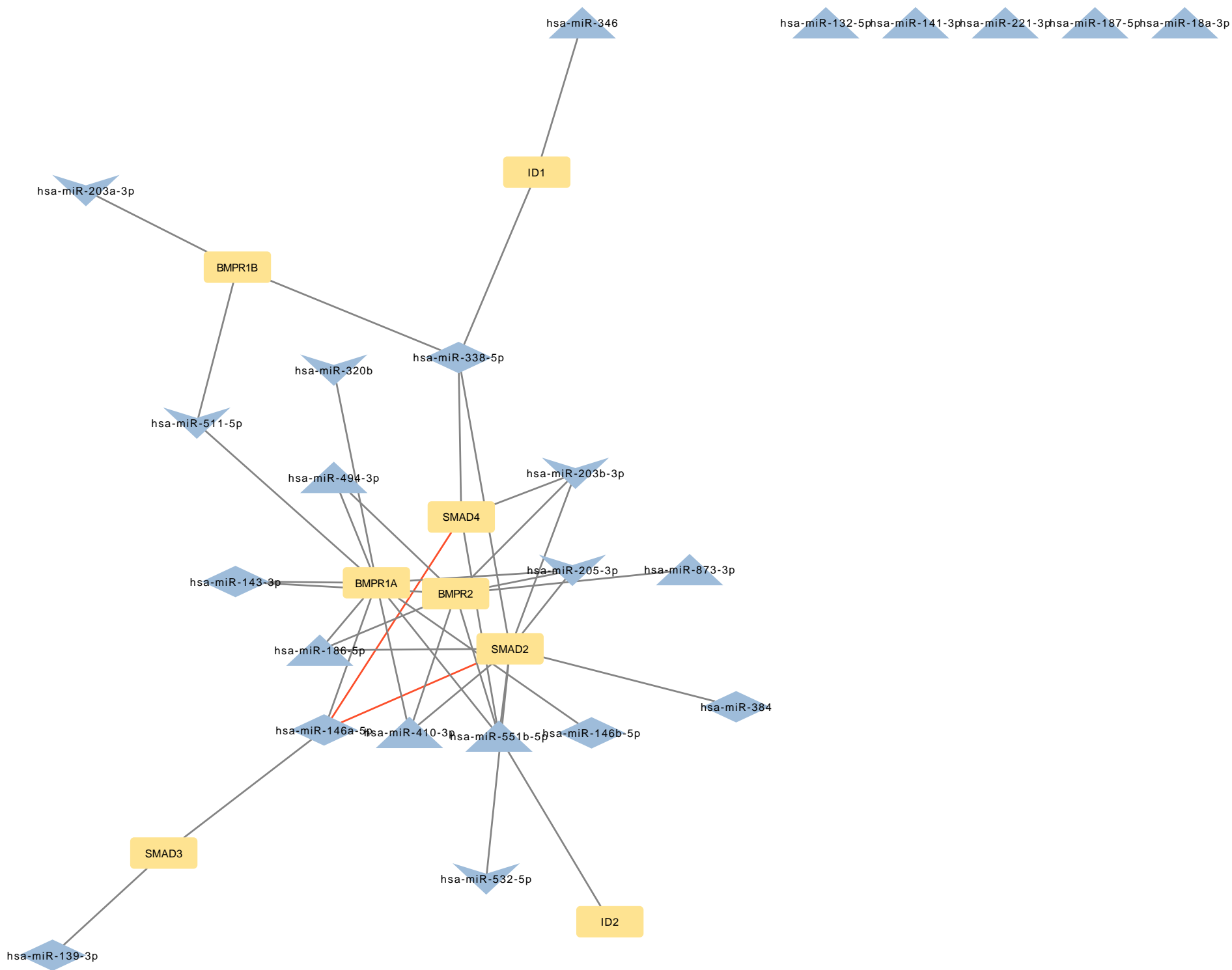

Supplement: Supplementary file 7 [file DataSheet_7.pdf]
